# Linkage Mapping of Stem Saccharification Digestibility in Rice

**DOI:** 10.1371/journal.pone.0159117

**Published:** 2016-07-14

**Authors:** Bohan Liu, Leonardo D. Gómez, Cangmei Hua, Lili Sun, Imran Ali, Linli Huang, Chunyan Yu, Rachael Simister, Clare Steele-King, Yinbo Gan, Simon J. McQueen-Mason

**Affiliations:** 1 Zhejiang Key Lab of Crop Germplasm, Department of Agronomy, College of Agriculture and Biotechnology, Zhejiang University, Hangzhou, China; 2 Centre for Novel Agricultural Products, Department of Biology, University of York, York YO10 5DD, United Kingdom; Institute of Botany, Chinese Academy of Sciences, CHINA

## Abstract

Rice is the staple food of almost half of the world population, and in excess 90% of it is grown and consumed in Asia, but the disposal of rice straw poses a problem for farmers, who often burn it in the fields, causing health and environmental problems. However, with increased focus on the development of sustainable biofuel production, rice straw has been recognized as a potential feedstock for non-food derived biofuel production. Currently, the commercial realization of rice as a biofuel feedstock is constrained by the high cost of industrial saccharification processes needed to release sugar for fermentation. This study is focused on the alteration of lignin content, and cell wall chemotypes and structures, and their effects on the saccharification potential of rice lignocellulosic biomass. A recombinant inbred lines (RILs) population derived from a cross between the lowland rice variety IR1552 and the upland rice variety Azucena with 271 molecular markers for quantitative trait SNP (QTS) analyses was used. After association analysis of 271 markers for saccharification potential, 1 locus and 4 pairs of epistatic loci were found to contribute to the enzymatic digestibility phenotype, and an inverse relationship between reducing sugar and lignin content in these recombinant inbred lines was identified. As a result of QTS analyses, several cell-wall associated candidate genes are proposed that may be useful for marker-assisted breeding and may aid breeders to produce potential high saccharification rice varieties.

## Introduction

Rice is one of the most important staple crops in the world, with the potential to feed more than two billion people [1, 2]. Asia, China and India produce and consume more than half of the world’s total rice supply [3]. Until recently, rice straw, which represents approximately half of the total biomass of the plant, was considered a waste stream of rice production with little or no value, depending on the country or region of production [4]. The practice of burning rice straw represents not only a waste of biomass resources, but also a series of challenges to the environment [5, 6]. In response to concerns about greenhouse-gas emissions and the sustainability of fossil fuel supplies, global biofuel production has expanded rapidly in recent years[7]. Many countries are starting to consider the use of rice straw for second generation biofuel production, and this previously underestimated lignocellulosic biomass is now viewed as a potential feedstock rather than a waste product [8].

Rice straw is composed mainly of lignocellulosic biomass, which is approximately 2/3 sugars with the potential for fermentation into biofuels[9]. However, the inherent recalcitrance of plant biomass to hydrolysis is an obstacle to saccharification [10]. This recalcitrance is conferred by the complexity and the crystallinity of the polysaccharides and lignin in the plant cell wall. Thus, facilitating the saccharification or improving the yield of digestible biomass could reduce the cost of industrial biofuel production [11]. Therefore, one approach is to screen rice varieties for cell wall components that are more susceptible to hydrolysis, without compromising the field performance of the crop [12]. There are many studies that focus on the molecular mechanism between cell wall polymers, chemotypes and biomass digestibility, and most of them demonstrate that altering either lignin content or the polysaccharide structure in the cell wall can affect saccharification potential of lignocellulosic biomass [13–18]. Linkage analyses in maize have shown that quantitative trait loci (QTL) associated with saccharification are independent of lignin abundance, suggesting that lignin is not the only factor determining saccharification potential[19]. Indeed, wider research has shown that changes in cellulose production, deposition, and crystallinity will also significantly affect saccharification [20–22]. Hemicelluloses are the second most abundant fraction in the cell wall, and they covalently link with lignin via ferulic acid ester linkages[23]. Changes in the structure of hemicelluloses, as well as the reduction of the covalent links with lignin can significantly affect saccharification of biomass[15, 24].

In order to move towards improved cell-wall digestibility, a RIL population derived from a cross between the lowland rice variety IR1552 and the upland rice variety Azucena was used in this study to research the genetic basis, candidate genes, and molecular markers that can be used in the identification of genes governing saccharification potential, to further the breeding of high saccharification rice varieties. These recombinant inbred lines were previously studied to analyze root architecture and cell wall expansion traits [25]. In the present work, we performed quantitative trait SNP (QTS) analyses with 271 molecular markers bases with saccharification potential, and further studied the relationship between reducing sugar and lignin content in these inbred lines. These results provide first hand genetic resources and potentially indispensable data for further research of cell-wall digestibility in *Oryza sativa*.

## Material and Methods

### Plant material

The RIL population was generated from a cross between IR1552, an irrigated lowland *indica* variety, and Azucena, an upland tropical *japonica* variety, by single-seed descent in the F_10_ generation (The seeds were kindly provided by Prof. Wu Ping). The rice plants were cultivated for two independent seasons (2012 summer and 2012 winter) in Changxing, Zhejiang Province (119°39' E, 30°54' N) and Sanya, Hainan province (109°31' E, 18°18' N) in China, respectively. There were no specific permissions required for these locations/activities, because all of the field test was conducted with permission of Zhejiang University's authority. The seedlings were arranged in single rows with 15cm spacing between plants and 30cm between rows, then they were grown under standard lowland rice field parameters [25]. Stem samples were taken between the third and the fourth node of mature and dried plants from these two independent seasons. All stem samples were cut into 4-mm lengths and ground using a custom-made robotic platform as previously described [26].

### Saccharification Analysis

Ground rice straw (4mg, 4 replicates per plant) was weighed into 96-deep-well plates, using a robotic platform [26]. A pre-treatment solution (0.5M NaOH, 350μL) was added and plates were sealed before incubation at 90°C for -30 min, followed by five washes with 500μL 25mM sodium acetate buffer, before incubation with 500μL of industrial enzyme mixture (solution of cellulases and hemicellulases) at 50°C with agitation. Aliquots(-45μL) of the resulting biomass hydrolysate were used for the determination of reducing sugars released by hydrolysis, using a modified 3-methyl-2-benzothiazolinone hydrozone (MTBH) method with robotic platform (Tecan Evo200; Tecan Group Ltd.), as previously described [26].

### Lignin content analysis

Ground rice straw was sieved to fine fraction (150 or 180μm sieve) before weighed into 200mg per plant, followed by 3 washes with 4mL CHCl_3_:MeOH (1:1) solution, 80% MeOH and 100%MeOH respectively (250rpm agitation for 30min, 10 min centrifuge at 2500g and 4°C), till powder turn into colorless. The washed powder were weighed to 3.5mg for each replicate before 4h incubation at 45°C with 0.3mL 25% (v/v) acetyl bromide/glacial acetic acid, then the mixture was cooled down to 15°C for adding 0.3mL glacial acetic acid, followed by a 5 min vortex. A 0.5mL of reaction mixture above were added to a tube containing 0.1mL of 0.5M hydroxylamine and 1mL of 2M NaOH solution. Tubes were gently shaken after acetic acid was added to give a final volume of 10 mL. The final solutions were read in spectrophotometer at 280 nm, the percentage of total lignin content in dry sample was calculated as L% = A_280_/17.2*6/5*10/3.5*100%.

### Association Mapping

The QTX Network software (http://ibi.zju.edu.cn/software/QTXNetwork/) was used to perform the quantitative trait SNP (QTS) analyses and dissect the genetic architecture of saccharification potential. Individual locus detection was conducted using a mixed linear model, and epistasis interacting loci were performed using a 2D genome scan with statistical model described before[27]. In total, 271 markers were used in the association mapping of 121 representative RIL plants (data from two seasons) with saccharification potential. Significant molecular markers were screened by the estimation of genetic main effects (additive A, dominance D) and prediction of epistasis effects (additive by additive AA, additive by dominance AD, dominance by dominance DD). The results were verified by estimation of superior genotypes (positive and negative) in general as well as in different environments and with reference to heritability for individual effects.

### Candidate genes mapping and localization

The candidate genes associated with QTS were selected by establishing the location of the marker in the rice genome and locating cell wall associated genes and transcription factors of selected superfamilies within a range 200Kb upstream and downstream of the marker using the Rice Genome Browser (http://rice.plantbiology.msu.edu/cgi-bin/gbrowse/rice/).

## Results

### Saccharification potential variation of RIL population.

**Fig 1:**
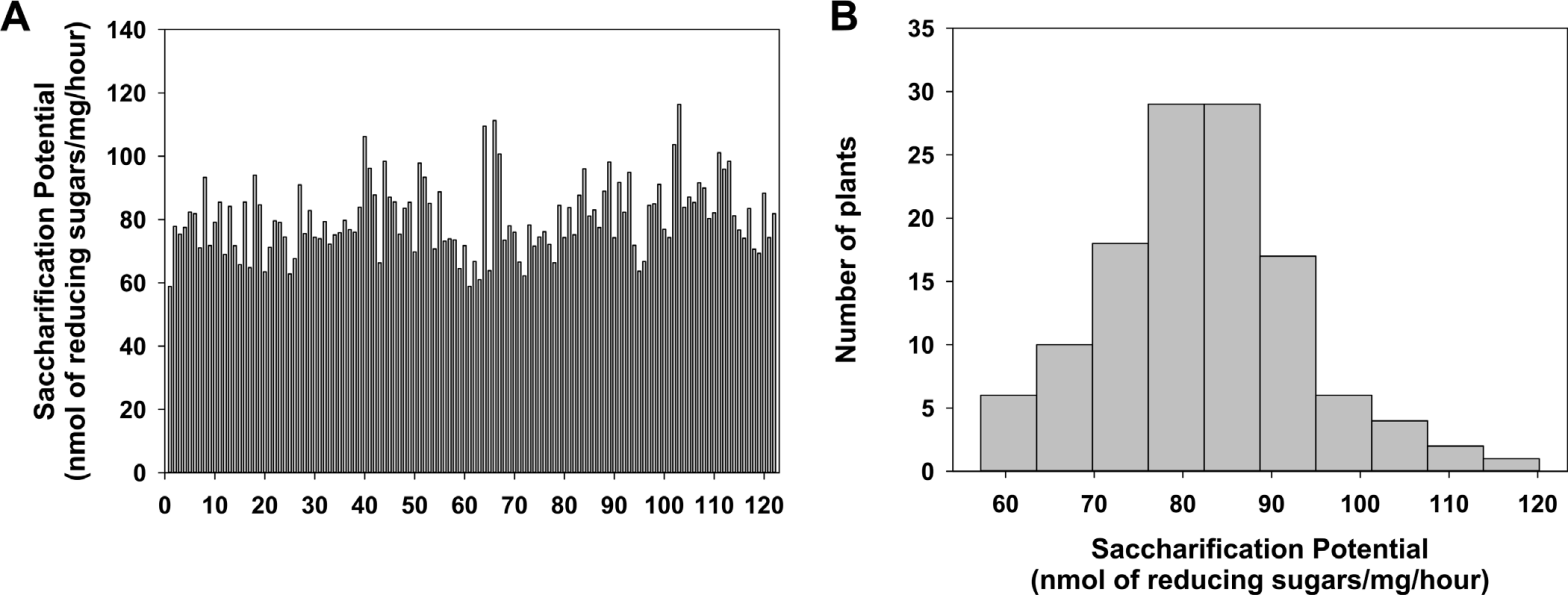
Sugar release from rice straws after 0.5M NaOH pretreatment at 90°C and hydrolysis of industrial enzyme mixture. (A) The saccharification potential (reducing sugar content) of 121 RIL lines. (B) Histogram showing normal distribution in saccharification of whole RIL population in first season dataset.

To understand further the mechanism behind the saccharification potential of rice straw, a population of 121 F_10_ RIL plants was grown to maturity, and the third internodes were collected to ascertain saccharification potential. The whole saccharification process was performed using a robotic platform, and the saccharification potential was measured by reducing sugar equivalents as described previously (Fig 1) [26]. Reliable results from the QTS studies require a wide distribution of the trait within the population. The saccharification data of the RILs from two seasons (four independent runs), shows a normal distribution across the population (Fig 1B) (P = 0.159, Shapiro-Wilk test), with a maximum/minimum reducing sugar content that is 55.87% higher / 25.81% lower than the median of the population, respectively. A wide range of saccharification values were observed, ranging from 59 nmol/mg.h to 116 nmol/mg.h, with the highest saccharification plant stems producing 97% more reducing sugar than the lowest saccharification line in this population. However, the two parental lines IR1552 and Azucena show 91.18% and 100.96% of the median, thereby showing a large diversity in saccharification potential in the 121 RIL population that we used in this study (Fig 1A & S1 Table).

### Lignin content is negatively correlated with saccharification potential

**Fig 2:**
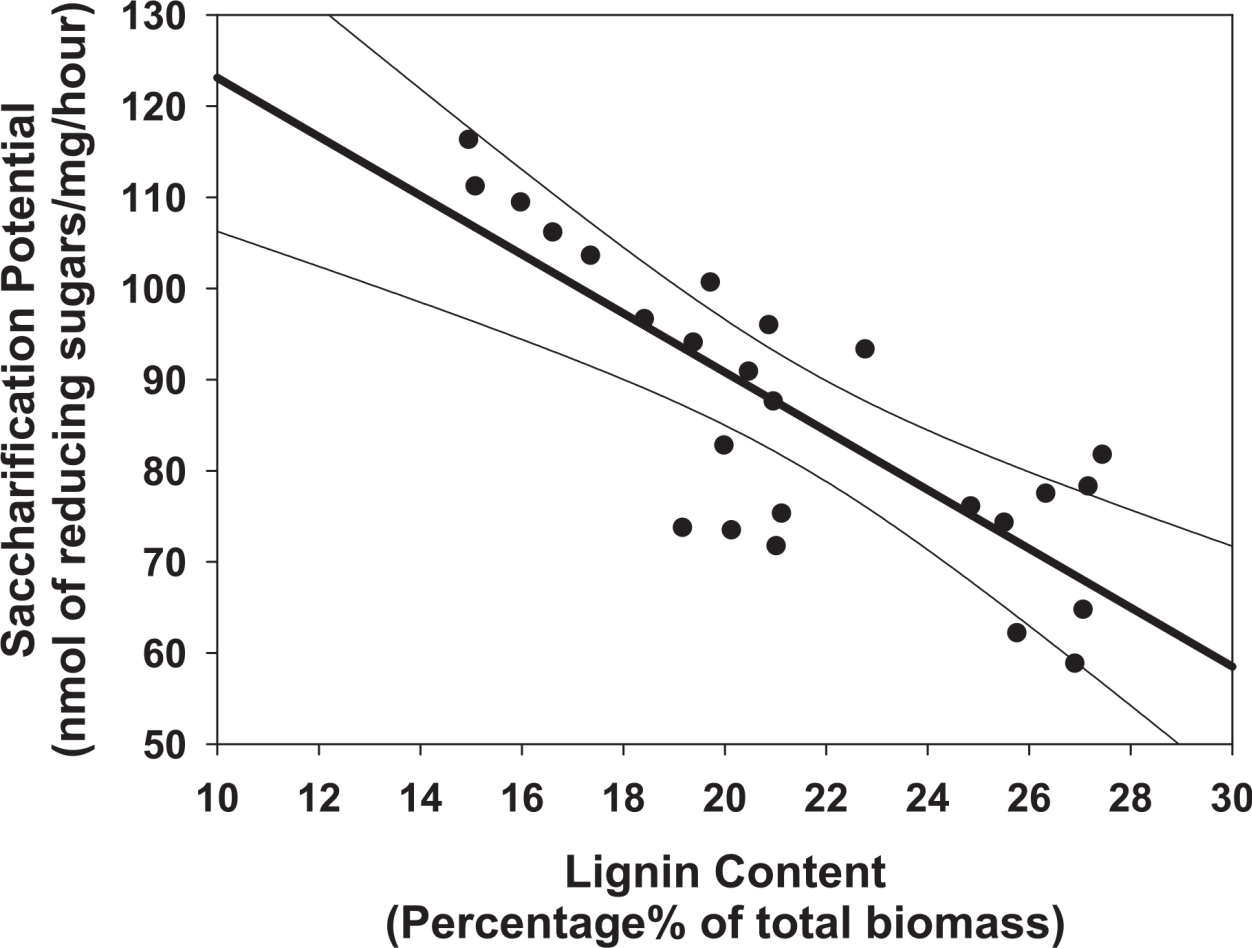
Lineal regression analysis of lignin content of 25 plants which have different saccharification potential from high to low. (a = 155.426, b = -3.231, R^2^ = 0.646; a, b and R^2^ represents the regression line intercept, the regression line slope and the coefficient of determination, 99% were used for confidence interval estimate)

In order to investigate how the cell-wall composition affects the saccharification potential, the lignin content of 24 different plants at different ends of the saccharification spectrum were analyzed by measuring the lignin content of the twelve lines with the highest/lowest yield of reducing sugars after hydrolysis. The results indicate that straw from lines with high saccharification potential has lower lignin content, suggesting that saccharification potential is negatively correlated with lignin content in rice (Fig 2). In lines with high saccharification (above 90 nmol/mg.h), the correlation between lignin and reducing sugars shows a tight linear correlation (r = 0.907, p<0.05), suggesting that high saccharification potential in rice straw is due mainly to low lignin content. Conversely, the data from rice lines with lower reducing sugar release (below 90nmol/mg.h) shows more variation in lignin content (r = 0.342, p = 0.231), indicating a more complex set of determinants of saccharification potential (19%< lignin content< 28%). Combined together, these results indicate that lines with high saccharification potential in this population were due, mainly, to a reduced lignin content, while higher recalcitrance is controlled by other cell wall features (Fig 2). This suggests that saccharification is a trait controlled by multiple functional genes combined together to modulate the composition and interaction of the cell wall polymers.

### QTS Loci Associated with Saccharification Potential

**Fig 3:**
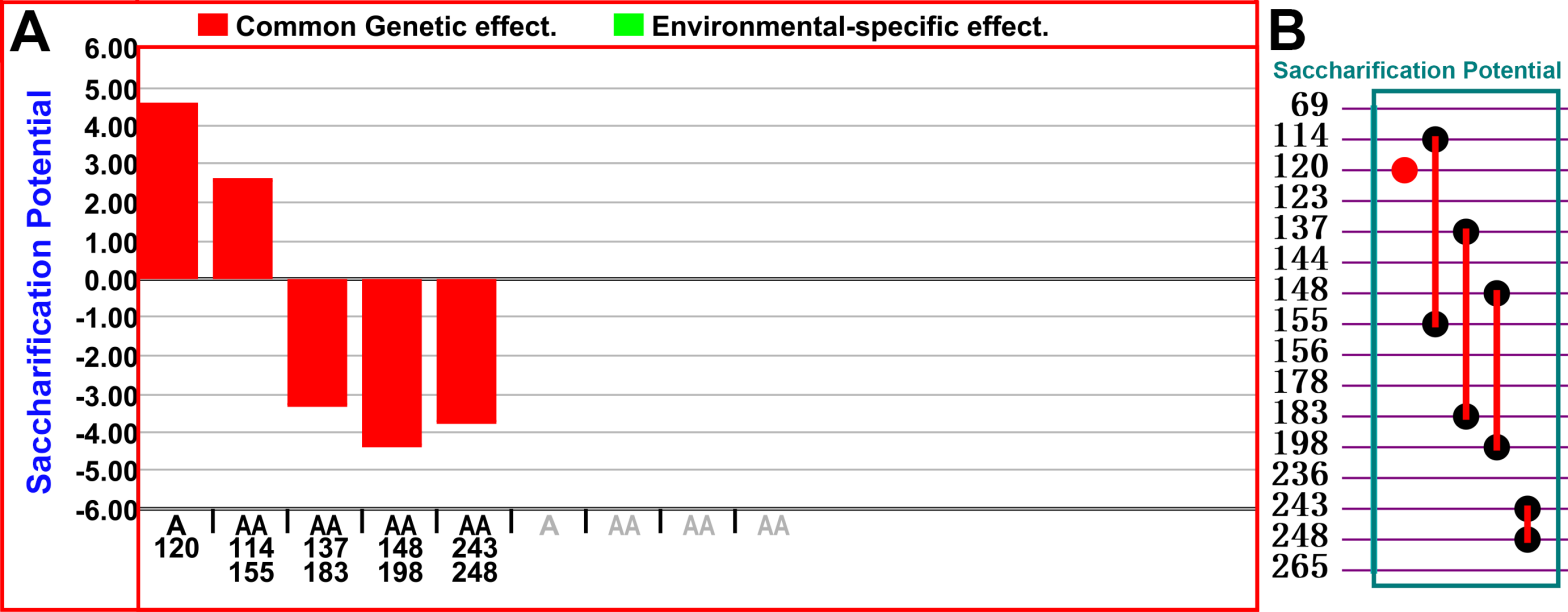
QTS linkage and association analyses of rice stem digestion potential. (A) GxE plot generated by QTX mapping. The left axis is the values of genetic effects, and the bottom axis is the SNP ID for loci; Red column = main effect, green line = environment-specific effect; A = additive effect; AA = additive-by-additive epistasis effect; (B) GxG plot generated by QTX mapping. Circle = additive effect locus; Line between two circles = epistasis effect of two loci; Red color = main effect; Black color = involving epistasis but with no individual locus effect.

The identification of QTS loci associated with saccharification potential requires the use of a population with a large number of markers. Among 121 lines, the population mean of reducing sugar equivalent is 78.36, with total heritability ($h_{G+GE}^{2}≜$ 40.32%). The genetic effect and genetic-environment interaction effect are large contributors to the saccharification potential in this population. In total, 271 markers, 1 phenotypic data with two environments and 121 individuals were used in this study. After analyses of two seasons of saccharification potential with marker set, one main additive effect locus RG264-120 ($h_{A}^{2}≜$ 11.78%, A = 4.45±0.65) and 4 pairs of epistasis loci (RZ70-114×RM72-155, $h_{AA}^{2}≜$ 3.80%, AA = 2.59±0.70), (AAG-CAG4-137×AGG-CAG11-183, $h_{AA}^{2}≜$ 6.14%, AA = -3.29±0.66), (RG146-148×RM144-198, $h_{AA}^{2}≜$ 10.71%, AA = -4.34±0.68), (AGG-CTG3-243×RG341-248, $h_{AA}^{2}≜$ 7.91%, AA = -3.73±0.71) were identified (S2 Table). The results demonstrated that there were no significant environment effects (Fig 3), which indicates that saccharification potential is determined, in the main, by genetic effect. The data suggested that only one main effect locus (RG264-120) and one pair of additive loci (RZ70-114×RM72-155) have a positive effect on the saccharification potential of this RIL population, and that three pairs of additive loci (AAG-CAG4-137×AGG-CAG11-183), (RG146-148×RM144-198), (AGG-CTG3-243×RG341-248) showed a negative effect on the saccharification potential of rice plants. These markers were distributed, in the main, on chromosomes 6,7,8,9,11 and 12 (Table 1)[25]. An investigation of the functional genes near these markers is required to provide more information on the detailed molecular mechanism behind these loci.

**Table 1:** Genetic position of Main QTLs Marker.

Marker No.	Name	Type	Chromosome	Genetic positon(cM)
114	RZ70	RFLP	8	60.00
120	RG264	RFLP	6	53.70
137	AAG-CAG4	AFLP	7	0.00
148	RG146	RFLP	7	154.70
155	RM72	SSR	8	44.30
183	AGG-CAG11	AFLP	9	186.90
198	RM144	SSR	11	17.60
243	AGG-CTG3	AFLP	12	170.30
248	RG341	RFLP	12	48.70

### Putative cell-wall associated gene associated with saccharification potential

The results presented indicate that lignin content is one of the main factors in establishing the saccharification potential of rice straw, especially in those lines with the highest digestibility. Traditional linkage analysis would provide QTL located between two markers within a centi-Mogan (cM) scale and this would cover hundreds or even thousands of candidate genes in the region, but exploiting the primer sequences and reference sequences of markers that were used in this study, and the availability of a reference genome in rice, it is possible to locate the exact position of the markers in the genome. Using these positions, cell wall associated genes in the region of the markers were identified (Table 2). (Due to the lack of information about the physical position of 4 of these markers, the candidate genes for 5 markers were investigated. Interestingly, 5 copies of UDP-glucoronosyl and UDP-glucosyl transferase genes were located close to only main effect locus (RG264). The glucosyl transferases may participate in the synthesis of polysaccharides during the cell-wall synthesis. The genes topologically associated with the other 4 markers, are involved in diverse cell wall processes in different cell wall fractions, and whilst. the proximity of these cell wall genes to identified markers does not represent a definite proof of their role in determining the saccharification potential, these candidate genes will be investigated further using molecular tools.

**Table 2:** Putative cell wall associated genes in the regions of the genetic markers linked to saccharification potential.

Marker^*^	Locus	Annotation
RZ70	Os05g41240	Myb-like DNA-binding containing protein
	Os05g41610	glucan endo-1,3-beta-glucosidase
	Os05g41760	AP2 domain containing protein
	Os05g41780	AP2 domain containing protein
	Os05g41870	glycine-rich cell wall protein
	Os05g41990	peroxidase extracellular
RG264	Os06g17090	UDP-glucoronosyl and UDP-glucosyl transferase
	Os06g17110	UDP-glucoronosyl and UDP-glucosyl transferase
	Os06g17120	UDP-glucoronosyl and UDP-glucosyl transferase
	Os06g17220	UDP-glucoronosyl and UDP-glucosyl transferase
	Os06g17260	UDP-glucoronosyl and UDP-glucosyl transferase
	Os06g17410	Dof zinc finger domain containing protein
RG146	Os07g43420	MYB family transcription factor
	Os07g43530	Helix-loop-helix DNA-binding domain containing protein
	Os07g43710	CSLA7—cellulose synthase-like family A
	Os07g43820	glycosyl hydrolase
	Os07g44070	pectinacetylesterase
RM72	Os08g34280	cinnamoyl-CoA reductase, CCR1
	Os08g34360	AP2 domain containing protein
	Os08g34900	pectinesterase
	Os08g34910	pectinesterase
	Os08g34960	MYB family transcription factor
RM144	Os11g47350	beta-D-xylosidase
	Os11g47390	laccase
	Os11g47460	MYB family transcription factor
	Os11g47500-610	glycosyl hydrolase
	Os11g47820	glucan endo-1,3-beta-glucosidase

*The physical position of Markers AAG-CAG4, AAG-CAG11, AAG-CTG3, and RG341 is not determined.

## Discussion

Rice straw represents an abundant source of lignocellulosic biomass, which is cheap and widely available in Asia. The utilization of this rice straw for the production of second generation biofuel would supplant the environmental problem of straw burning with an environmentally favorable alternative that also reduces dependency on fossil fuels. However, the recalcitrance of rice straw to digestibility is a significant barrier to the realization of large scale industrial conversion of rice straw into biofuel. Lignin has been widely considered as the major determinant of cell-wall digestibility, and in poplar, alfalfa and tobacco, it has been demonstrated that down-regulation of catalases involved in lignin synthesis pathway can lead to a reduction in lignin content and an increase in saccharification compared with wildtype [28–31]. However, it has also been established that the modification of cellulose, matrix polysaccharides, or cell architecture will also affect the saccharification of lignocellulosic biomass[13, 15, 22, 24, 32]. Nonetheless, lignin appears as the main determinant of the saccharification potential only in rice lines with low lignin content. The negative correlation between lignin content and saccharification potential in lines with lower lignin content is not observed when lignin content is high, supporting the hypothesis that whilst lignin content is a key factor, it is not the only obstacle preventing the hydrolysis of polysaccharides. The saccharification potential of cellulosic biomass is also affected by transcription factors that modify the expression of genes directly involved in the modification and biosynthesis of cell wall content. A group of secondary wall NACs (*SND1*, *VND6/7* and *AtNST1-3* in Arabidopsis[33–35], PtrWNDs in poplar[36], *MtNST1* in Medicago[37]) and MYBs (*AtMYB46/83*, *AtMYB58/63*, *AtMYB61* and *AtMYB85* in Arabidopsis[38–42], *ZmMYB31*, *ZmMYB42* and *ZmMYB46* in maize[43, 44], *OsMYB46* in rice[45]) transcription factors have been well characterized. As secondary wall and lignin associated transcription factors, the SWNs (Secondary wall NACs) can bind to an 19bp consensus sequence called SNBE (Secondary wall NAC binding element), which is widely found in promoters of cell wall biosynthesis and modification genes[45]. The SWMs (Secondary wall MYBs), involved in the regulation of lignin biosynthesis were found to be expressed specifically in the metaxylem. [46]. Taken together, the SWNs and SWMs present a complex and coordinated regulatory network between lignin biosynthesis and other plant physiological pathways. In addition, transcription factors in other superfamilies were also found to be involved in the regulation of lignin biosynthesis. These include *PtrWRKY19* in *Populus trichocarpa*, *PpDof5* in *Pinus pinaster*, *SbbHLH1* in *Sorghum bicolor*, *EjAP2-1* in *Eriobotrya japonica*, *BpMADS12* in *Betula platyphylla Suk*. Since digestibility is a trait determined by genes that affect the physical structure and chemo-types of the secondary cell-wall, finding markers and identifying the genes involved in conferring recalcitrance is a major step towards improving biomass feedstocks.

In a study of the genetic basis of high saccharification potential in rice, a QTS analysis of a RIL population that was generated from a cross between IR1552 and Azucena was performed. This population has been used previously for root traits under different water supply conditions [25]. After saccharification analyses of rice straw from this population during two independent growth seasons, the saccharification data showed a normal distribution, within a large range of values. This indicates that the cross between IR1552 and Azucena generated widely and randomly distributed values for saccharification potential and will be a useful tool to find markers associated with this trait. Further QTS analyses demonstrate that there is one main additive effect locus (RG264-120) and one pair of additive loci (RZ70-114×RM72-155) that can enhance the saccharification potential in this RIL population, and three pairs of additive loci (AAG-CAG4-137×AGG-CAG11-183), (RG146-148×RM144-198), (AGG-CTG3-243×RG341-248) that can reduce the saccharification potential of these rice plants. These results demonstrate that there are more interacting loci pairs involved in the modification of saccharification potential than single dominant loci. This may due to the distant consanguineous relationship between IR1552 and Azucena even the saccharification of the two parents were not deviate far from the median of the population. In contrast the lowest and highest individuals showed a much wider deviation from median, suggesting that the new combination of interacting locus generated by the hybridization of this two varieties may contribute most to this phenomenon. In the F10 generation of hybrid progeny, most loci were well segregated, but few QTLs locus were detected, due to the density and type of molecular markers in this population. Nonetheless, it was established that the genetic location and sequences of these markers are widely distributed in the rice genome, in combination with reference genome databases, our results provide new genetic information regarding the combined regulation of saccharification potential.

Based on annotation of genes adjacent to these loci candidate genes with a role in cell wall and lignin synthesis were selected. (Table 2). Among the 39 cell wall associated candidate genes that were selected some showed tandem copies. Among the candidates, the glucan endo-1,3-β-glucosidases (Os05g41610, Os11g47820) have been associated with cell expansion processes [47, 48].The glycine-rich cell wall protein (Os05g41870) has been reported to influence the plasticity of the cell wall, and is expressed in xylem and phloem, playing an important role in early cell wall development [49–51]. *OsPRX74* (Os05g41990) is an extracellular peroxidase that might be involved in the biosynthesis and degradation of lignin, and has also been reported to be involved in responding to stress and pathogens[52–54]. *OsCSLA7* (Os07g43710) is a cellulose synthase-like A family member genes, and may play an important role in mannan synthesis, mannan being a component of the non-cellulosic polysaccharides in cell-walls [55, 56]. *CCR1* (Os08g34280), encodes a Cinnamoyl-CoA reductase as one of the key enzymes involved in the biosynthesis of monolignols and downregulation of its expression can lead an enhanced saccharification potential in *Medicago sativa*[28]. Pectin acetylesterase (Os07g44070) is involved in pectin deacetylation in primary cell wall of higher plants, which will influence the composition and structure of cell wall polysaccharides [57, 58]. UDP-glucoronosyl and UDP-glucosyl transferase (Os06g17090, Os06g17110, Os06g17120, Os06g17220, Os06g17260) are glycosyltransferases, that have been associated with the modulation of glycosylation and polymerization of polysaccharides during secondary cell wall formation processes [59, 60]. Pectinesterases (Os08g34900, Os08g34910) are involved in pectin modification during fruit softening and cell wall pectin metabolism[61, 62]. Beta-D-xylosidase (Os11g47350) takes part in remodeling processes [63, 64]. Laccases (Os11g47390) are a key component of the lignin synthesis process[65]. Glycosyl hydrolases (Os11g47500-610, Os07g43820) are part of a large family involved in cellulose and hemi cellulose degradation[66]. Furthermore, there are also 4 MYBs (Os05g41240, Os07g43420, Os08g34960, Os11g47460), 3 AP2s (Os05g41760, Os05g41780, Os08g34360), 1 bHLH(Os07g43530) and 1 Dof-like (Os06g17410) transcription factors found near the QTLs markers, but their function and role in the regulation of lignin and cell wall biosynthesis remains unknown.

The direct involvement of these candidate genes in altering the cell wall recalcitrance requires further research, including a study of the interaction of these genes and epistatic effects observed. Although QTS studies fall short of establishing the genes involved in cell wall recalcitrance, the results presented provide initial and valuable information on markers to assist in the selection of more digestible rice straw, which will be of benefit for further breeding of high saccharification potential rice varieties and their utilization for sustainable biofuel production.

## Supporting Information

S1 Table Saccharification Data of RILs in two independent seasons. (XLSX)

S2 Table Report of QTXNetwork Software. This is a detailed report exported from QTX analysis with QTXNetwork Software.
(XLSX)
